# Combination of Estradiol with Leukemia Inhibitory Factor Stimulates Granulosa Cells Differentiation into Oocyte-Like Cells

**DOI:** 10.34172/apb.2021.080

**Published:** 2020-07-26

**Authors:** Soudabe Yousefi, Maryam Akbarzadeh, Jafar Soleimanirad, Kobra Hamdi, Laya Farzadi, Aalie Ghasemzadeh, Mahdi Mahdipour, Reza Rahbarghazi, Mohammad Nouri

**Affiliations:** ^1^Stem Cell Research Center, Tabriz University of Medical Sciences, Tabriz, Iran.; ^2^Department of Biochemistry and Clinical Laboratories, Faculty of Medicine, Tabriz University of Medical Sciences, Tabriz, Iran.; ^3^Student Research Committee, Tabriz University of Medical Sciences, Tabriz, Iran.; ^4^Stem Cell and Regenerative Medicine Institute, Tabriz University of Medical Sciences, Tabriz, Iran.; ^5^Department of Applied Cell Sciences, Faculty of Advanced Medical Sciences, Tabriz University of Medical Sciences, Tabriz, Iran.; ^6^Women’s Reproductive Health Research Center, Tabriz University of Medical Sciences, Tabriz, Iran.

**Keywords:** Granulosa cells, Leukemia inhibitory factors, Follicle-stimulating hormone, Estradiol, Oocyte-like cells

## Abstract

***Purpose:*** Previous studies have documented that cumulus granulosa cells (GCs) can trans-differentiation into different non-ovarian cells, showing their multipotentiality to repopulate the injured cells in ovarian tissue. The current experiment is aimed to assess the differentiation capacity of human cumulus GCs toward the oocyte-like phenotype *in vitro*.

***Methods:*** GCs were isolated from healthy female volunteers subjected to *in vitro* fertilization or intra-cytoplasmic sperm injection (IVF-ICSI). The effect of different media supplemented with leukemia inhibitory factors (LIFs), 5 ng/mL estradiol, and 0.005 IU/mL follicle-stimulating hormone (FSH) were investigated to the differentiation of GCs toward oocyte-like phenotype via monitoring the expression of Oct3/4 and GATA-4 using flow cytometry analysis. The expression of genes such as *FIGLA*, *NOBOX*, and *SYCP3* was measured by real-time polymerase chain reaction (PCR) assay. We also assess morphological adaptation by using bright-field microscopic imaging.

***Results:*** Exposure of GCs to LIFs increased the number of cells expressing stemness factor Oct3/4 coincided with the suppression of GATA-4 after 7 days (*P* < 0.05). We found that the transcript level of all genes *FIGLA*, *Nobox*, and *SYCP-3* decreased in cells after treatment with a FSH (*P* < 0.05). According to our data, the incubation of GCs with estradiol increased the expression of genes related to the oocyte-like phenotype.

***Conclusion:*** Our finding revealed that the combination of LIFs and estradiol could induce the GCs’ oogenesis capacity and thereby is possibly suggested as a therapeutic strategy during the occurrence of gynecological disorders.

## Introduction

Infertility is a multifactorial disorder and induced by numerous environmental factors such as changes in lifestyle and nutritional habits.^1^ Some conventional techniques such as *in vitro* fertilization/intra-cytoplasmic sperm injection (IVF-ICSI) are commonly used to circumvent the complications after the onset of infertilities. Unfortunately, conventional approaches do not always contribute to reliable results. Therefore, alternative approaches with the potential to improve IVF-ICSI efficiency are extensively under investigation.^2^ Female infertility is originated from a variety of endogenous reasons such as hypothalamic dysfunction, premature ovarian failure, polycystic ovarian syndrome or early menopause, and tube fallopian insufficiency. Nevertheless, the absence or reduction of follicles within ovaries and oogenesis suppression is thought of as the most leading cause of infertility. Recently, ovarian progenitor cells and their differentiation toward oocyte-like cells have been of great interest to restore the ovarian tissue competence.^3-5^ In mammals, oocytes are surrounded by a large number of granulosa cells (GCs) layers during folliculogenesis. After entering the prenatal stage, theca cells envelop the follicle containing oocytes. Theca cells in collaboration with GCs produce estrogen to provide structural integrity and maintain the synthesis of androgen substrates.^6^ The existence of stem cells in follicular theca and ovarian epithelium was previously reported. For instance, Hubner et al revealed an inherent capacity of embryonic stem cells trans-differentiation into oogonia with normal meiosis. These cells were also able to recruit neighboring cells to generate follicle-like unites and blastocysts.^7^ In another study done by Bukovsky and colleagues, the differentiation capacity of ovarian tunica mesenchymal cells toward epithelial-like cells was found. These cells could pass through ovarian blood vessels, and play as ovarian germ cells. In the next steps, these cells participate in the reformation of follicles in adult women post-puberty.^8^ Further studies on a variety of pluripotent cells showed that GCs are capable to express stem cell markers.^9^ In 2012, Yamanaka proposed four factors OCT4, SOX2, KIF4, and c-MYC as pluripotent stem cells markers.^10^ Later, the expression of these factors in GCs was shown in various studies, indicating the suitability of these cells for reprogramming.^11,12^ Considering the stemness feature of GCs and ease of extraction during surgery and biopsy procedure, it is noteworthy to mention that a large GCs number could be achieved for *in vitro* culture systems. Here, we aimed to investigate the oocyte-like differentiation of GCs after exposure to leukemia inhibitory factors (LIFs), follicle-stimulating hormone (FSH), and estradiol (E_2_).

## Materials and Methods

### Granulosa cells expansion

In this study, follicles were sampled by transvaginal ultrasound-guided aspiration from the women patients who referred to the infertility clinic for ICSI procedure. All candidates signed the informed consent form. Inclusion criteria were regular monthly ovulation, the mature oocytes, and normal values of thyroid and sex hormones. Patients with the history of polycystic ovaries, ovulation disorders, dysfunction in sex hormones, and HIV also CMV diseases were excluded from the study. Follicular fluid (FF) was aspirated by an expert embryologist, and then the GCs surrounding oocytes were separated and transferred into a sterile falcon tube containing an ISM1 medium. After a quick spin, the cells were washed twice with sterile phosphate-buffered saline (PBS) and centrifuged at 1200 rpm for 10 minutes to remove FF. Then, cells were cultured as described in the next section. The aspirated FF was also centrifuged at 3000 rpm for 10 minutes, inactivated at 56˚C for 45 minutes and filtered. The filtered liquid was stored at -20°C until used.

### Cell culture

**Figure 1:**
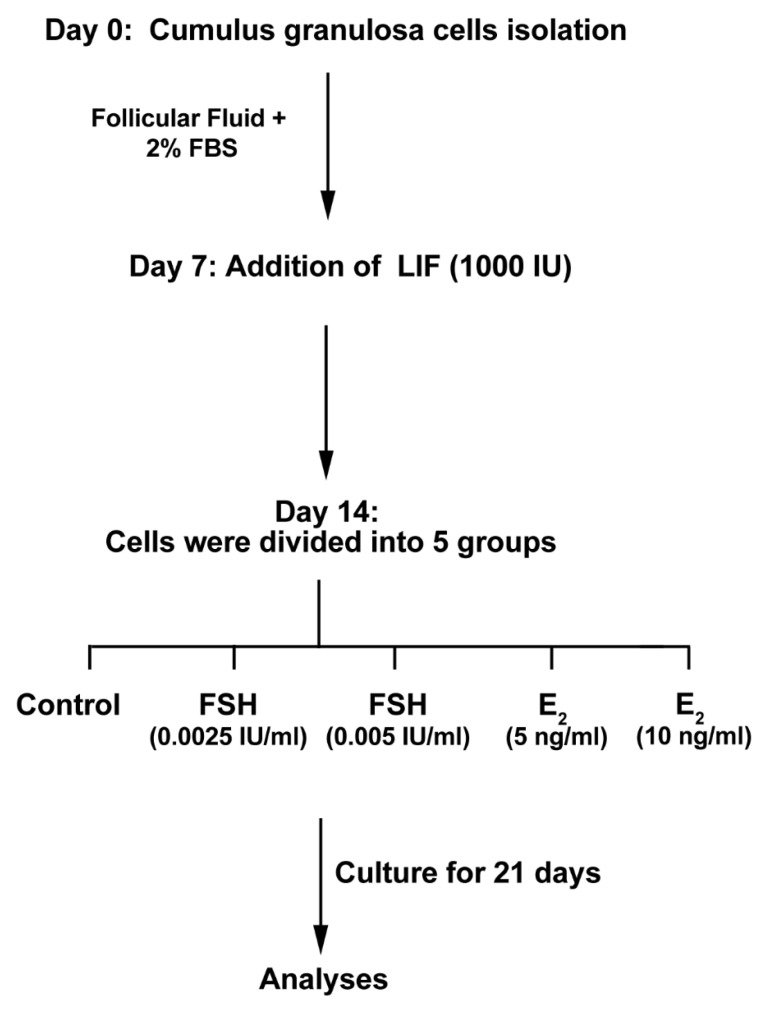
Diagram of the experimental procedure of cumulus granulosa cells exposed to leukemia inhibitory factor (LIF) and estradiol (E_2_), and follicle-stimulating hormone (FSH) throughout 35 days to assess differentiation potential toward oocyte-like cells.

GCs were expanded in DMEM/F12 medium (Gibco) containing 10% FF, 2% fetal bovine serum (FBS; Gibco) and %1 penicillin-streptomycin (Sigma) solution. 3×10^5^ cells/well were placed in each well of 6 well-plates (TPP) and maintained at 37˚C in a humidified atmosphere and 5% CO_2_. Culture media were replenished every 48 hours. In the second week, GCs were incubated with 1000 IU LIF (Peprotec) to induce a stem cell-like phenotype. From the beginning of the 3^rd^ week, cells were incubated in DMEM/F12 medium supplemented with 10% FF, 2% FBS, 10 ng/mL basic fibroblast growth factor (bFGF; Sigma), 10 µL/mL non-essential amino acids (Invitrogen), 0.1 µL/mL retinoic acid (Sigma), 10 ng/mL epidermal growth factor (EGF; Sigma) and 1 mM 2-Mercaptoethanol. In this study, cells were then divided into five main groups as follows (Figure 1); (I) Control, (II) cells received 0.0025 and 0.005 IU/mL FSH and III: cells were incubated with 5 and 10 ng/mL E_2_. The medium was replaced every 24 hours and the cells were passaged over 5 weeks.

### Detecting E2 receptor (ER) by immunofluorescence assay

For this purpose, 10^4^ GCs were placed in each well of 8-well slide champers. After 24 hours, GCs were fixed by using a pre-cooled paraformaldehyde solution (4% w/v). Then, cells were incubated in 1% Triton-X100 solution (Sigma-Aldrich) for 5 minutes for permeabilization. To detect ER, rabbit anti-human ER (dilution: 1:500; Abcam) and Texas Red-conjugated antibody (Dilution: 1:2000, Abcam) were used. For nuclear staining 200 µL, DAPI (1 µg/mL) was poured onto wells and the slides were imaged using fluorescence microscopy.

### Flow cytometry analysis

The flow cytometry analysis was done to confirm the existence of stem cell-specific marker Oct3/4 (BD) and granulosa cell-specific marker GATA4 (BD). To this end, 7 and 14 days after treatment with different factors, cells were detached from the plates using 0.25% Trypsin-EDTA solution (Gibco). Permeabilization was performed by using the TritonX-100 solution (0.1% w/w). A panel of primary antibodies including Oct3/4 and GATA4 was used. Appropriate fluorescent secondary antibodies were applied for cell staining. The BD FACSCalibur^TM^ system and FlowJo software (ver.7.6.1) were used to perform flow cytometry analysis. This experiment was used in triplicate.

### Real-time PCR assay

Expression of *FIGLA*, *NOBOX*, and *SYCP3* was evaluated using real-time polymerase chain reaction (PCR) assay. On day 14, the whole RNA was extracted by using an RNX PLUS Kit (Cinnagen, Iran). The content of RNA was measured by a NanoDrop (2000c spectrophotometer; Thermo Fisher). We used the cDNA synthesis kit (Bioneer) to synthesize cDNA. The real-time PCR analysis was performed by Corbett Rotor-Gene 6000 machine (Corbett Life Science) in a final volume of 14 µL reaction system containing 0.8 µL of each primer (Table 1), 7 µL of SYBR green reagent (Takara Bio, Japan), 0.8 µL of cDNA template, and nuclease-free water.

**Table 1:** Primer list

**Gene**	**Primer sequence**	**Accession number**	**Annealing (°C)**
*GAPDH*	F: 5´AAGCTCATTTCCTGGTATGACAACG-3´	NM_002046.3	58
	R: 5´TCTTCCTCTTGTGCTCTTGCTGG-3´		
*FIGL A*	F: 5´-CCAAGGAGCGTGAGCGGATAA-3´	NM_001004311.3	60
	R: 5´-TACTATAGCTCTGCTCATCTGG-3´		
*NOBOX*	F: 5´-CTGATGGATGTTGCTGGCAGTGA-3´	NM_001080413.3	59
	R: 5´-AAGGGGAAAGTGGGGAGGTAGGG-3´		
*SYCP*_3_	F: 5´-CTCAGAAGCGTCGCGGAGAAG-3´	NM_001177948.1	61
	R: 5´-CTTCCGCAATGGCCGAGGACCAG-3´		

### Statistical analysis

All experiments were done in three independent replicates. Results were reported as mean ± SD. Statistical analyses were performed using GraphPad Prism (version7.0). Significant differences were calculated using a one-way analysis of variance (ANOVA) and a Student *t*test. The mean difference between the data was significant at the level of *P  <  0.05, **P  <  0.01 and *** *P*  <  0.001.

## Results and Discussion

### Immunofluorescence staining showed typical markers in cultured granulosa cells

**Figure 2:**
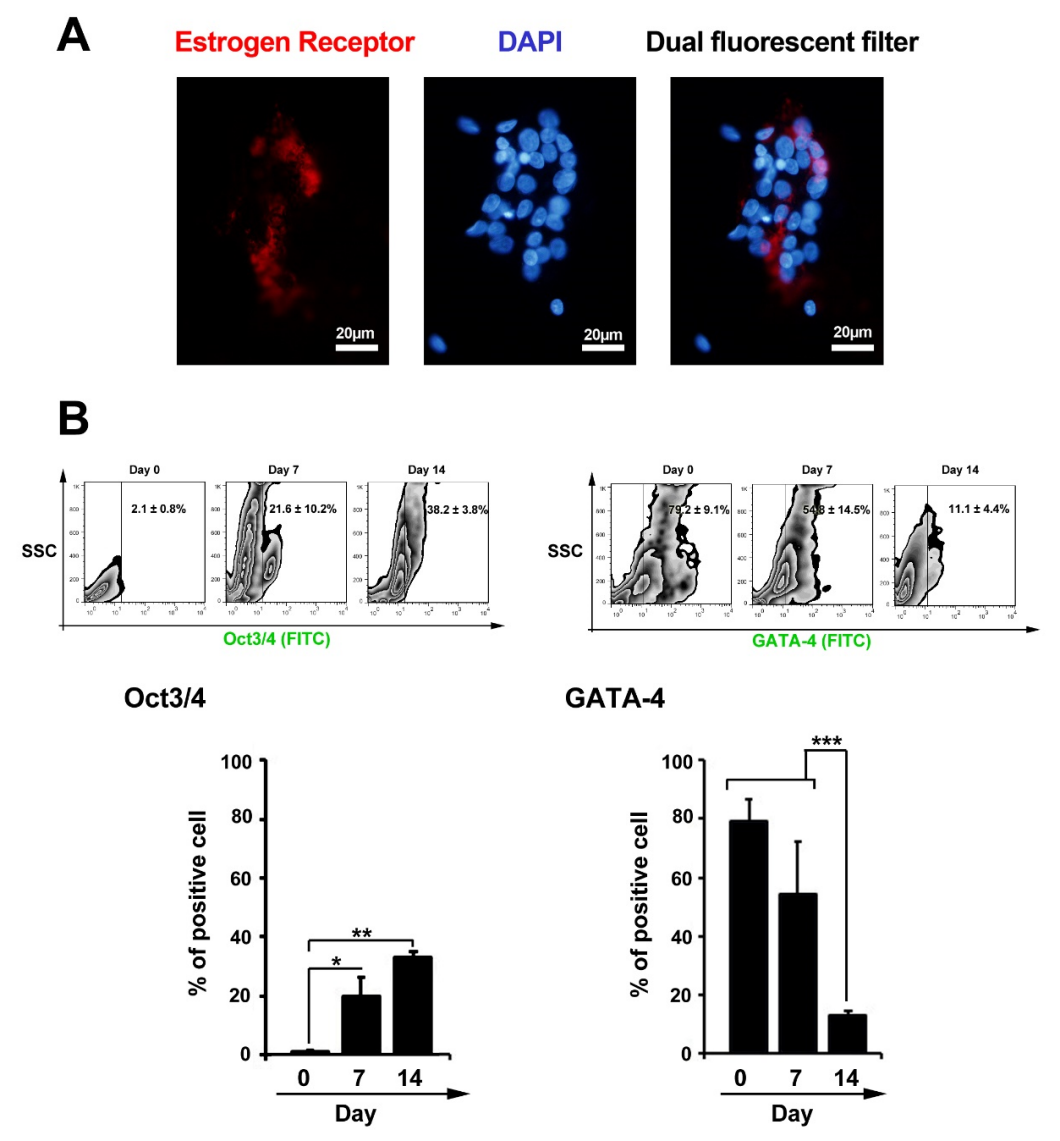
Measuring estrogen receptors in cultured granulosa cells using immunofluorescence staining (**A**). Data showed a dim expression of estrogen receptors in granulosa cells at passage three. Flow cytometric analysis of Oct3/4 and GATA-4 in granulosa cells 7 days after treatment with leukemia inhibitory factor (**B**). Data showed statistically significant induction and reduction of Oct3/4 and GATA-4 in granulosa cells, respectively (n=3); One-way ANOVA with Tukey post hoc test *P  <  0.05; **P  <  0.01 and ***P  <  0.001).

We examined the existence of ER in GCs using IF imaging (Figure 2A). As presented in Figure 1A, the cellular distribution of ER was identified in GCs obtained from human samples. Here, we showed a dim expression of ER in cultured GCs. These results showed that GCs had the potential to express ER, indicating an inherent cell ability to respond to E_2_.

### Changes in the levels of Oct3/4 and GATA-4 in granulosa cells treated with leukemia inhibitory factor

Flow cytometry analysis showed that GCs could express Oct3/4 14 days after incubation with LIF (Figure 2B). Compared to non-treated cells at initial seeding time, the percent of Oct3/4 positive GCs were reached 38.2 ± 3.8% on day 14 (*P*_Day 14 versus Day 0_ < 0.01; *P*_Day 7 versus Day 0_ < 0.05). These data confirm stemness-like features in GCs after exposure to the LIF. Besides, the level of GATA-4 reached to minimum levels, from 79.2 ± 9.1% to 11.1 ± 4.4%, at the end of incubation time (*P*_Day 14 versus Day 0_ < 0.001; Figure 2B). We also found a significant drop in the level of GATA-4 coincided with the induction of Oct3/4 in GCs. These data showed the potential of LIF to induce multipotentiality in the GCs. Parte et al first reported the expression of the pluripotent genes, including STAT-3, NANOG, Oct-4, TERT, and Sox-2, in the ovarian epithelium.^13^ Virant-Klun et al identified adult stem cells in the human ovaries with the ability to trans-differentiate into oocyte-like cells and form parthenote-like complexes.^14^ Studies pointed multipotent stem cells could commit to germ cell lineages and functional gametes by applying numerous differentiation methodologies, including the addition of LIF or FF to the culture medium with ovarian GCs.^15-18^ LIF, as one of the most studied pro-pluripotency factors, promotes the self-renewal by activating various signaling pathways such as STAT3 and BMP4 and MAP kinase pathways.^19^ This factor is secreted from the outside of the fetus, as well as many other types of mature cells such as endometrial cells, fibroblasts, bone cells, monocytes, macrophages, and T lymphocytes. LIF initiates intracellular signaling pathways after binding to receptors LIFR and gp130.^18,20,21^ The cell distribution of the CD29, POU5F1, CD90, CD44, CD166, CD105, and CD117 factors was reported on the surface of GCs by Kossowska-Tomaszczuk et al.^22^

### Real-time PCR analysis showed up-regulation of oocyte-related genes

**Figure 3:**
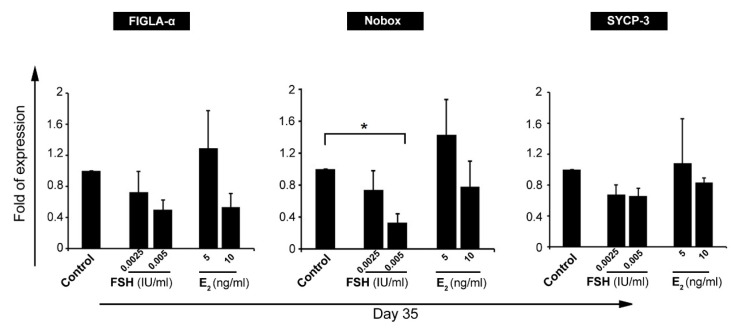
Real-time PCR analysis of oocyte-related genes, *FIGLA-α, Nobox*, and *SYCP-3*, in granulosa cells after exposure to follicle-stimulating hormone (FSH) and estradiol (E_2_) (*n*=3). Gene expression analysis confirmed the significant reduction of *Nobox* gene in granulosa cells after treatment with FSH. These data showed the potency of FSH in inhibition granulosa cell differentiation toward oocyte-like cells.

Real-time PCR analysis showed the up-regulation of *FIGLA-a*, *Nobox*, and *SYCP-3* in GCs treated with 5 ng/mL E_2_ and these effects were less in groups received 10 ng/mL E_2_. According to these data, it seems that E_2_ enhanced oocyte-like stemness in GCs in a certain dose (Figure 3). In contrast, data showed that FSH treatment, at doses 0.0025 IU and 0.005 IU per ml, suppressed the activity of *FIGLA-a*, *Nobox*, and *SYCP-3* compared to the control cells (Figure 3). *In vitro* studies have further demonstrated that GCs are capable of differentiation into lineages of neurons, osteoblasts, and chondrocytes which are not observed in normal ovarian follicles.^22^ Varras et al showed that DAZL mRNA, a typical germ cell marker, was not detectable in GCs, suggesting that GCs are not originated from primordial germ cells^12^. The maintenance of GCs stemness is one of the most important and considerable issues in the trans-differentiation of the GCs into another cell lineage.^23^

### Morphological adaptation was shown in granulosa cells after exposure to estradiol and follicle-stimulating hormone

**Figure 4:**
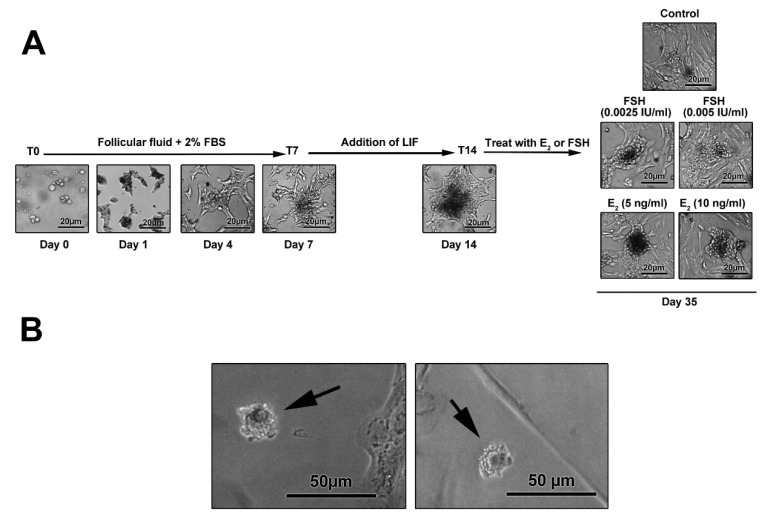
The morphological adaptation of granulosa cells cultured in different media supplemented with leukemia inhibitory factor (LIF), estradiol (E_2_), and follicle-stimulating hormone (FSH) after 35 days. Cell morphology in E_2_-treated cells (**B**). After the completion of the experimental protocol, cells lose epithelial-like appearance and acquire a round shape. The cell spreading and flattening are confined and the extent of projection decreased following treatment with LIF and E_2_. It seems that the up-regulation of oocyte-like genes, such as *Nobox*, in granulosa cells coincides with prominent morphological adaptation.

Photomicrographs showed the ability of freshly isolated GCs to attach the bottom of the culture. GCs exhibited an epithelial-like appearance after 7 days post-seeding (Figure 4A). The addition of LIF to culture medium generated micro-aggregates and colonies. Using FSH and E_2_, colonies became more compact and large gaps were evident between the colonies (Figure 4A). On day 35, single oocyte-like cells were observed with a round shape (Figure 4B). It has been elucidated that FF contains various factors secreted from GCs, theca cells and oocytes, and plasma, namely GDF9b, GDF9, stem cell factor (SCF), bFGF, and E2.^24,25^ These factors are involved in the regulation of follicular development.^24^ In a study conducted by Virant-Klun et al, the culture of epithelial cells in a medium with FF generated round-shaped-cell clusters with alkaline phosphatase activity and primitive oocyte phenotype and up-regulation of SOX-2 and SSEA-4.^26^ We, here, showed that the exposure of GCs to E_2_ and FF contributed to the formation of colonies and oocyte-like cells. These cells can synthesize factors OCT4, SOX-2, etc., which are known pluripotency markers of stem cells.^17^ Dyce et al tested several culture systems to identify conditions in which porcine skin-derived sphere cells could differentiate into germ-cells. They documented that FF advocates the induction of markers expression coincided with germ-cell differentiation.^17^ These data support the notion that the combination of FF with the appropriate factor levels could promote GCs orientation to different lineage, especially oocyte-like cells. In line with this statement, some previous studies documented that FSH and E_2_ have beneficial effects on the antrum-like reorganization, proliferation, differentiation as well as endocrine function of the GCs.^27-29^ Unlike these results, we did not find any changes in the level of oocyte-associated markers after cell exposure to FSH. Robker and Richards showed that E_2_ and FSH can directly and independently regulate the process of the cell cycle in GCs by increasing levels of cyclin D2.^29^ However, there is not enough evidence about the effects of the FSH and E_2_ on the trans-differentiation capacity of GCs into oocyte-like cells.

## Discussion

Nowadays, a lot of research is done to address the molecular mechanisms causing the low quality of oocytes.^13,30^ Identification of a subpopulation of GCs to exhibit a pluripotent and self-renewing potential opens new horizons in augmenting new therapeutic strategies for patients suffering from ovarian insufficiencies.^22,26^ We showed that the culture of GCs with LIF increased the expression of OCT3/4 while down-regulated GATA-4 in GCs. Additionally, the treatment of GSs with FSH diminished the expression of oocyte-related genes. E_2_ can promote the expression of *FIGLA-a*, *Nobox*, and *SYCP-3*. These data support the notion that E_2_ could efficiently preserve the stemness characteristics of the GCs as compared to FSH-treated cells.

## Conclusion

In conclusion, our study showed that the exposure of the GCs to the LIF and E_2_ efficiently preserves the GCs multipotentiality by inducing the expression of oocyte-like cell genes. This approach offers a novel strategy in the medication of infertility and ovary restoration in the treatment of gynecological disorders.

## Ethical Issues

All experiments and procedures were conducted in compliance with the ethical principles of Tabriz University of Medical Science, Tabriz, Iran (TBZMED.REC.1394.100). This study was supported by a grant from Tabriz University of Medical Sciences.

## Conflict of Interest

None declared.

## Acknowledgments

The authors kindly thank Dr. Shaneband to design primers.
